# Immunogenetic, Molecular and Microbiotic Determinants of Eosinophilic Esophagitis and Clinical Practice—A New Perspective of an Old Disease

**DOI:** 10.3390/ijms221910830

**Published:** 2021-10-07

**Authors:** Alina Kanikowska, Szymon Hryhorowicz, Anna Maria Rychter, Marcin A. Kucharski, Agnieszka Zawada, Katarzyna Iwanik, Piotr Eder, Ryszard Słomski, Agnieszka Dobrowolska, Iwona Krela-Kaźmierczak

**Affiliations:** 1 Department of Gastroenterology, Dietetics and Internal Diseases, University of Medical Sciences, Przybyszewskiego 49, 60-355 Poznan, Poland; a.m.rychter@gmail.com (A.M.R.); marcinku@ump.edu.pl (M.A.K.); aga.zawada@gmail.com (A.Z.); piotr.eder@op.pl (P.E.); agdob@ump.edu.pl (A.D.); krela@op.pl (I.K.-K.); 2 Institute of Human Genetics, Polish Academy of Sciences, Strzeszynska 32, 60-479 Poznan, Poland; ryszard.slomski@up.poznan.pl; 3 Department of Pathomorphology, University of Medical Sciences, Przybyszewskiego 49, 60-355 Poznan, Poland; katarzyna.iwanik@ump.edu.pl

**Keywords:** eosinophilic esophagitis, genetic factors, environmental factors, molecular mechanism, microbiome, diet therapy

## Abstract

Eosinophilic oesophagitis (EoE) is a chronic, allergic disease associated with a T-lymphocyte response inducing esophageal eosinophilic infiltration in the esophagus. Inflammation and tissue fibrosis are responsible for the main clinical symptoms such as food impaction and dysphagia. The etiopathogenesis is multifactorial in which genetic and environmental factors coexist. The most common trigger is a non-IgE-mediated food allergy to milk, wheat, egg, soybean, nuts, fish, and seafood. The second factor we focus on is the contribution of genetic variation to the risk of EoE, describing the expression profile of selected genes associated with eosinophilic oesophagitis. We raise the topic of treatment, aiming to eliminate inflammation through an elimination diet and/or use of pharmacologic therapy with the use of proton pump inhibitors or steroids and endoscopic procedures to dilate the esophagus. We demonstrate that early diagnosis and effective treatment prevent the development of food impaction and decreased quality of life. The increasing presence of EoE requires bigger awareness among medical specialists concerning clinical features, the course of EoE, diagnostic tools, and management strategies.

## 1. Introduction 

Eosinophilic esophagitis (EoE) is an inflammatory process of different layers of the esophagus driven by antigen-mediated mechanisms involving Th2 cells response leading to eosinophil migration to esophageal tissue. EoE is considered the leading cause of dysphagia and food impaction in adults and children. The incidence of EoE is growing due to the increasing frequency of allergies and better diagnostic tools but still is underdiagnosed and undertreated. The entity is rather new; it was established as a clinically distinct syndrome around 30 years ago [1]. Patients with EoE not only suffer from physical symptoms such as heartburn, abdominal pain, dysphagia but also have a low quality of life and altered eating behaviors such as avoidance of eating with other people, fear of suffocation, limited list of foods, slow chewing, or an increased fluid intake with meals. The prevalence of EoE ranges from 5 to more than 80 cases per 100,000 inhabitants depending on the assessment method. In the United States Dellon et al. estimated that prevalence of EoE occurs in 56.7/100,000 persons [2]. In one of the studies by Dellon et al., EoE was observed in 2–7% of patients who had endoscopy for any reason, and 12–23% with endoscopy due to dysphagia [1]. The age of onset may vary from the very first months of life to adult years; there are two peaks observed: one in children between 5 and 10 years of life and a second in adults between 30 and 50, with the predominance in Caucasian male patients three times more common than in female patients [2,3,4]. There is a difference between symptoms reported by women who suffer mainly from chest pain and heartburn and men, who complain of dysphagia and food impaction [5]. Confirmation of EoE diagnosis is based on an endoscopic sampling of the esophagus with an eosinophil count higher than 15 per high power field in at least one biopsy. Etiopathogenesis of EoE is multifactorial, in which genetic, immunological, and environmental factors play roles leading to impaired epithelial barrier function and Th-2-dependent inflammation. There are emerging two phenotypes of the disease; one is coexists with atopic diseases, the other with non-allergic mechanisms such as in patients with connective tissue disorders [6]. This article aims to present an updated insight into immunogenetic, molecular, and microbiotic factors predisposing to EoE along with the clinical course of the disease and treatment [7].

## 2. Pathogenesis of Eosinophilic Esophagitis

The pathogenesis of EoE is still not clear, there is genetic susceptibility observed, but rapid increase in morbidity over the last decades, also due to better diagnostic tools, seem to be mainly acquired they to environmental factors as they correspond with the increasing frequency of allergic diseases. Understanding modifiable factors could help prevent and treat EoE more effectively, but so far despite the growing number of studies concerning etiopathogenesis-gathered data is not precise and sometimes conflicting.

### 2.1. Environmental Factors

EoE occurs more often in patients with other allergic diseases such as atopic dermatitis, Ig-E mediated food allergy, allergy to aeroallergens, asthma, and allergic rhinitis. It is considered one of the diseases taking part in allergic march [8,9,10].

The incidence of allergic and autoimmune diseases has increased dramatically over the years in developed countries, and EoE is among them. Environmental factors that predispose to that situation are premature birth, caesarian section, neonatal intensive care admission, maternal fever, early antibiotic, and proton pump inhibitors (PPI) exposure, formula feeding, lack of contact with diverse microbiotic exposure—“hygiene hypothesis”, a Western diet with a high amount of fatty acids, all of which may lead to dysbiosis in the digestive tract and altering of immune system function. In the experimental study of EoE by Silva et al., obesity induced by high-fat diet led to increased inflammation in the esophagus and higher remodeling area comparing to the esophagus in lean mice with EoE [11,12,13]. The role of the microbiome in EoE is discussed in detail in a further part of the article.

Infectious diseases may predispose to EoE, such as *Herpes Simplex* virus infection by damaging esophageal mucosa, which can be a trigger for eosinophilic inflammation [14]. Observed in recent years, lower incidence of *H. pylori* in children was also a factor suspected to enhance EoE development, but this correlation was not confirmed by Molina-Infante et al.’s study conducted in 23 centers [15].

Contrary to the allergy predisposing factor—urban residency, Jenssen et al. presented a study in which EoE was more common in patients from rural areas [16].

Oral food immunotherapy (OIT) is a risk factor for EoE, especially in patients with Ig-E mediated allergy to milk, egg, and shellfish. Whether OIT induces EoE or only exacerbates already existing mild inflammation needs to be confirmed [17]. Further studies need to analyze if IgE-mediated food allergy, even after developed tolerance, does not pose a risk factor to EoE triggered by the same food but in non-IgE-dependent mechanisms.

A few studies showed that in some patients, both adults and children, symptoms of EoE appear or aggravate in the pollen season, in spring and/or summer [18,19]. Mishra et al. found that sensitization to aeroallergen by respiratory tract promotes esophageal eosinophilia in a murine model [20].

It is important to diagnose EoE in patients who are allergic to pollens, especially those who are treated with sublingual immunotherapy (SLIT) to pollens because EoE is a contraindication to this type of immunotherapy. The possibility exists that, in some patients, SLIT itself provokes EoE. Thus far, there are several case studies published presenting the development of EoE symptoms after initiation or maintenance SLIT therapy to pollens or dust mites, which tend to resolve after discontinuation of treatment [21,22,23].

Among many factors that can be associated with the pathogenesis of EoE, we can also find several behavioral aspects which seem to be important. First of all, the consumption of alcohol can negatively affect the risk of EoE. In the study by Koutlas et al., current alcohol consumption—regarding 75% of EoE cases—was moderately associated with EoE; however, after multivariate analysis, this association was not observed [24]. In the Lipka et al.’s study, both tobacco and alcohol used by non-treated patients were associated with a higher risk of severe stricture when compared to the individuals not using tobacco or alcohol [25]. However, the risk of EoE through smoking is not fully known, as other studies have shown that tobacco use reduced the risk of EoE [26]. Furthermore, Slae et al. investigated if breastfeeding duration—associated with other allergic and atopic diseases—can be an early life exposure risk factor among children; however, they did not find a statistically significant influence of breastfeeding practices on EoE risk [27]. On the other hand, IgE-mediated food allergies (especially for peanuts and tree nuts) were observed more frequently among children with EoE. At this point, it is essential to highlight that although non-IgE-mediated food allergy is well established among the pathogenesis of EoE—also in adults—it should be remembered that not all allergens can increase the risk in the same way or the same proportion [28]. It should be also further investigated how and if the presence of IgE-mediated food allergy influences the diagnosis or the course of the disease [1,10,29]. Moreover, other environmental factors, for example, contaminated crops or livestock with bad quality (treated with hormone and antibiotics) can also increase the risk of EoE, mostly by affecting other risk factors for EoE, e.g., microbiota dysbiosis [30].

### 2.2. Immunogenetics

The histopathological hallmark of EoE is the presence of eosinophils in the hyperplastic oesophageal epithelium. Straumann et al. first hypothesized an allergic origin of EoE by showing that T cells express IL-5 and immune cells contain IgE in oesophageal biopsy specimens from EoE patients [31]. The assumption of a key allergic factor causing EoE was confirmed by animal model studies, where it was shown that eosinophilic infiltration in the esophagus is associated with cytokines, eotaxins, IL5, IL 13, and other Th2 mediators. A 2017 study by Kottyan and colleagues showed that immune-related Th2 signaling pathways, particularly those involving interleukin 4 and 13, are critical to the initiation and pathoetiology of EoE [32]. It appears that, during active disease in humans, interleukin 13 is repeatedly induced in the oesophageal cells leading to the secretion of an important chemokine (eotaxin 3) [33] and an oesophageal-specific proteolytic enzyme (calpain 14) [34]. The former, involving eosinophils, is responsible for the remodeling and accumulation of collagen, the latter for the disruption of the oesophageal epithelial barrier by dislodging desmoglein-1 (DSG 1). Thus, IL-13 decreases the production of DSG 1, thereby increasing the alteration of the oesophageal epithelial barrier [35,36]. Furthermore, it turns out that, in mice, overexpression of IL-13 is already sufficient for the appearance of eosinophilic infiltration in the esophagus and other structural changes characteristic of EoE. In addition to the loss of epithelial barrier integrity and Th2-related immune responses, the oesophageal tissue is profoundly altered, with the frequent presence of mucosal rings, strictures, linear furrows, and trachealization [37]. Immunogenic determinants of EoE risk will predominantly be variants in genes associated with Th2 signaling pathways such as *CRLF2* (Cytokine Receptor-Like Factor 2), *CCL26* (encodes eosinophil chemoattractant eotaxin 3, chemokine C-C motif ligand 26)—which is the most upregulated gene in EoE [38] *FLG* (encodes filaggrin), *DSG1* (desmoglein-1) *STAT6* (Signal transducer and activator of transcription 6)—which is involved in the regulation of eotaxin 3, calpain 14 and desmoglein, *CAPN14* (calpain 14) and *TSLP* (Thymic Stromal Lymphopoietin) overexpressing in the oesophageal epithelium and activating Th2 lymphocytes [36].

Candidate gene association studies allow the identification of genes and signaling pathways suspected to be involved in the conditioning and pathogenesis of a given disease. Determining the frequency of single alterations (small mutations or SNPs) in the genes studied, between patients and controls, can provide extremely valuable information on the etiology of the disease. However, in the case of searching for new, previously unidentified variants, this method is associated with a certain limitation. It is therefore important to note that EoE is characterized by a highly conservative gene expression profile [38]. Thus, for assessing the genetic variability of EoE risk loci, the results obtained from GWAS analyses could be highly valuable. However, it should be noted that the identification of molecular mechanisms influencing disease risk is not an easy task since over 90% of genetic variants associated with immunological and allergic diseases, such as EoE, are located in non-coding regions [32,39,40,41,42]. The actual establishment of a causal relationship in non-coding regions is a tough call, and it is important to realize that it may depend on specific cell types and the presence of specific inflammatory signaling pathways.

GWAS results reveal that, in EoE patients, various alterations are common in the region (5q22) associated with the *TSLP* and *WDR36* genes [43]. GWAS studies have shown one significant EoE-associated locus in a region 5q22 associated with *TSLP* and *WDR36* genes [43,44,45]. Over time, studies of other candidate genes showed an association of EoE susceptibility also with the TSLP receptor: *CRFL2* (cytokine receptor-like factor 2), *FLG* (filaggrin), and *CCL26* (eotaxin 3) which was overexpressed about 50-fold compared with controls which have considerably strengthened its role in EoE pathogenesis [38,46]. However, all these variants together with the 5q22 region were also characteristic of other atopic conditions [47,48]. Other GWAS studies have identified two genes as important in the pathogenesis of EoE: the *CAPN14* on chr2p23.1 and the *EMSY* gene on chr11q13.5 [42,44,49]. Genome-wide association studies demonstrate that specific genetic associations between EoE and epithelial-related genes may play key roles in driving the Th2-type inflammation typical of EoE as shown in Table 1.

As our understanding of the pathogenesis of EoE increases, it is logical to anticipate that in the future further EoE risk loci will be identified, e.g., tissue—as so far defined *CAPN14*, or *the ANKRD27*, *PDCD5* and *RGS9BP* genes [32], possibly affecting the expression of the surrounding one or more genes in the esophagus through direct effects or modulation of chromatin structure. It will also certainly be possible to identify EoE risk loci other than TSLP and WDR36 associated with other allergic phenotypes and to identify molecular mechanisms driving genetic linkage in EoE. Statistical analysis of causal variants and genotype-dependent transcriptional analysis is needed to identify potential disease mechanisms.

### 2.3. Microbiotic Factors

The microbiota is an essential element to consider when discussing gastrointestinal disorders. Progress in microbiota research has been accelerating in recent years. In healthy individuals, Streptococcus group bacteria are mainly isolated from the esophagus. Whereas, in active inflammation, Gram-negative anaerobic or microaerophilic bacteria predominate [50]. In addition, the oral and esophageal microbiome of children with EoE is richer in Proteobacteria (Neisseria and Corynebacterium). Gram-positive Streptococcus and Atopobium predominate in healthy children [51]. The hygiene hypothesis responsible for the development of EoE causes changes in commensal microorganisms, which increase serum immunoglobulin E (IgE) and basophil responses [52]. Other risk factors for allergic diseases, such as cesarean section, antibiotic exposure, and lack of breastfeeding and neonatal intensive care unit stay, alter immune tolerance and redirect the effects of the commensal microbiota to stimulate the T helper-2 (Th2) lymphocyte phenotype [53,54]. Eosinophil accumulation in the esophagus secretes defensins and has a stunning effect on bacterial cell DNA, affecting the local microbiota [55,56]. The microbiota of untreated EoE patients showed a shift from a predominantly Gram-positive population to an increase in Gram-negative Haemophilus and Proteobacteria [50]. In a study by Benitez et al., analysis of the bacterial composition of oral swabs and esophageal biopsies by 16S rRNA sequencing showed an increase in Proteobacteria (Neisseria and Corynebacterium) in the group with EoE. In contrast, in the control group without EoE, bacteria of the Firmicutes family predominated [51]. The elimination of allergens from the diet did not result in significant differences in the microbiota, whereas their reintroduction enriched the esophageal biota with Campylobacter and Ganulicatella species previously described as characteristic of various chronic inflammatory conditions [51,57,58] In contrast, a study by Harris et al. showed a significant increase in Haemophilus in untreated EoE patients [59] Hiremath et al. analyzed saliva samples from 19 control children without EoE and 26 children with EoE by sequencing the 16S rRNA; they found a trend toward lower microbial richness in children with EoE. In contrast, the amount of Haemophilus species bacteria was significantly higher in active EoE than inactive EoE and increased with eosinophilic esophagitis’s increasing histological scoring system. This correlation may indicate Haemophlius quantification as a noninvasive marker of EoE activity [60]. Fecal microbiota is also altered in EoE. In a study by Kashyap et al., fecal microbiota was assessed in 12 patients with EoE and 12 controls by 16SrRNA amplification. Patients with EoE showed significantly lower diversity of gut microbiota. The authors observed a significant increase in Bacteroidetes, and decrease in Firmicutes, and a significant reduction in Clostridiales and Clostridia in patients with EoE [61]. This fact is essential in the context of studies that have shown that Clostridia groups protect against the development of food allergies in rodents [62] H. pylori infection increases the expression of interferon-gamma and interleukin-17, resulting in the proliferation of Th1 and Th17 cells and a consequent reduction in the number of Th2 cells associated with atopy [63,64]. Eradication of H. pylori and reduction in infections with this bacterium correlates with a rapid increase in the incidence of EoE, [65,66]. The protective effect of H. pylori against the development of atopic diseases is also confirmed by other animal studies [67,68]. A meta-analysis of 11 observational studies found that exposure to H. pylori compared to no exposure was associated with a 37% reduction in the chance of developing EoE [69]. However, the Molina-Infante study questions this relationship [15].

Probiotic supplementation may also have an impact on the course of eosinophilic esophagitis. The probiotic Lactococcus lactis NCC 2287 is a potent inhibitor of the eosinophil survival cytokine IL-5 [70]. By this, it could reduce the severity of food allergy. Holvoet et al. tested in a mouse model whether supplementation with L. lactis NCC 2287 and B. lactis NCC 2818 reduces histologic symptoms of EoE. The study showed that sensitized mice receiving L. lactis NCC 2287 had significantly less esophageal eosinophilia than the non-sensitized group. This fact was attributed to the effect of L.lactis NCC 2287 in reducing levels of IL-5L, which is a potent inducer of EoE [71].

Consideration of the influence of the microbiota is therefore essential when analyzing patients with EoE.

## 3. Diagnostic Standards of Eosinophilic Esophagitis

Clinical symptoms are the main reason to perform endoscopic examination of the upper gastrointestinal tract. Usually, adult patients complain of chest pain, abdominal pain, dysphagia or odynophagia, regurgitation, nausea, anorexia, while in children emesis, refusing eating, failure to thrive and abdominal pain are the main complaints [72].

Diagnosis of EoE should be based on the correlation between clinical features, endoscopic impression, and microscopical finding of biopsy material obtained during endoscopy [73].

The gold standard of diagnosis is based on histopathologic examination of biopsies taken during esophagogastroduodenoscopy. It is recommended to take six to eight biopsy samples from the distal and proximal part of the esophagus, due to the patchy nature of esophageal eosinophilia. In up to 25% of patients, the endoscopic pattern is normal [74].

Just after biopsy the tissue samples have to be preserved in 10% buffered formaldehyde. Routine preparation and hematoxylin and eosin (HE) staining are enough for slides.

**Figure 1:**
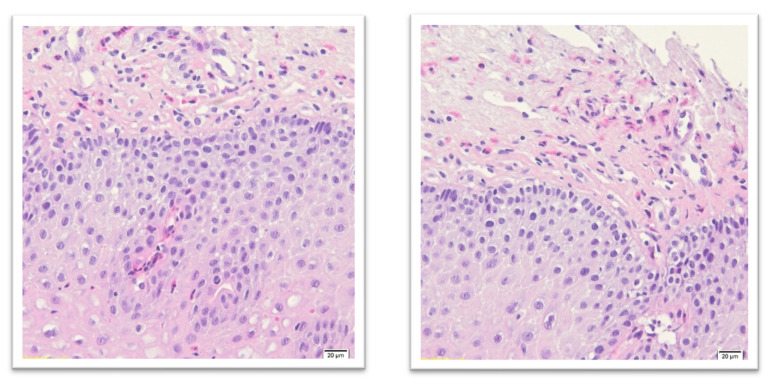
Histopathological images with HE staining showing esophageal eosinophilia (our own source: collection of the Pathomorphology Department).

A peak eosinophil count of 15 eosinophils or more per high-power field (HPF 40) in at least 1 of the standard size of ~0.3 mm^2^ samples allows suspicion of EoE [75]. Histopathological images with HE staining showing esophageal eosinophilia are presented in Figure 1. The presence of eosinophils in the esophagus is a nonspecific finding, not an absolute criterion for the diagnosis of EoE [73]. Moreover, it is suggested that there is not absolute cut-off number of eosinophils differentiating EoE from other eosinophil-rich conditions, mainly from GERD [73].

Other features very important and helpful in supporting the diagnosis of EoE by Feakins [73] are:

- Epithelial layer expanded and pale;
- Squamous cells vacuolization and spongiosis with visible desmosomes;
- Basal layer expansion;
- Papillary elongation;
- Surface disrupted with squamous cell necrosis (dense eosinophilic band);
- Eosinophils concentration (usually 30 and more/HPF) near the epithelial surface;
- In superficial epithelium eosinophilic microabscesses (more than four contiguous eosinophils);
- Eosinophil degranulation with intracellular eosinophilic dust;
- Variable submucosal hyaline fibrosis;

Despite the morphological aspect presented above, many patients have peripheral eosinophilia (3).

There are other histology-based scoring systems, e.g., the Eosinophilic Esophagitis Histology Scoring System (EoEHSS) developed by the Collins assessing grade and stage for eosinophilic inflammation, epithelial basal zone, eosinophil surface layering, eosinophil abscesses, dilated intercellular spaces, and lamina propria fibrosis [76,77].

Another system, the Anti-Eosinophil Peroxidase Monoclonal Antibody-Based Histopathologic Scoring System also enables identification of samples suspected of EoE that did not reach 15 eosinophils per HPF, with the possibility of degranulated eosinophils assessment. This system additionally helps to differentiate EoE from GERD with esophageal eosinophilia [78].

**Figure 2:**
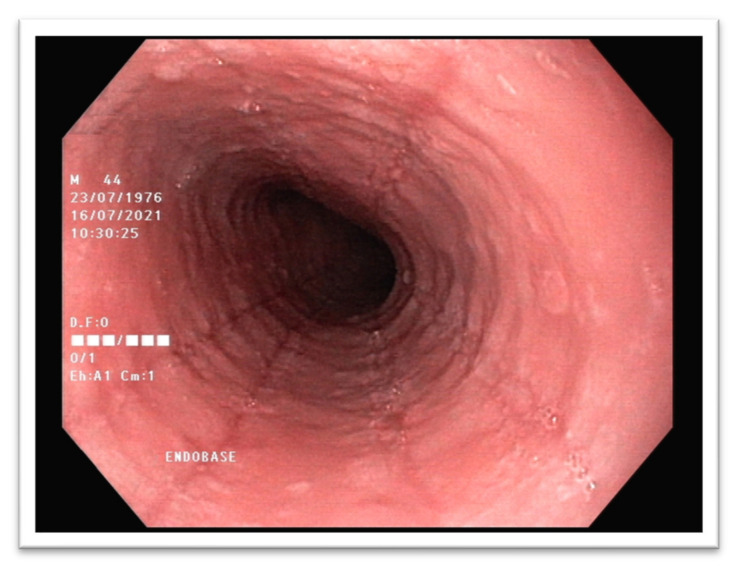
Endoscopic image of EoE showing linear furrows. (Our own source: collection of the Gastroenterology Department).

**Figure 3:**
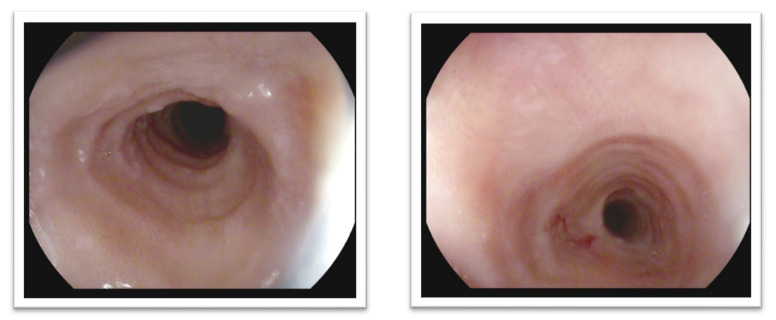
Endoscopic images of EoE showing esophageal rings (trachealization) and mucosal fragility. (Our own source: collection of the Gastroenterology Department).

There are certain characteristic features such as exudates as white specks, edema, linear furrows, rings, strictures, or crepe-paper esophagus that may indicate EoE in endoscopy. There is an endoscopic scoring system developed called EREFS (acronym for edema, rings, exudates, furrows, stricture) that is a standardized tool to assess endoscopic EoE disease activity. Each feature is graded separately. Edema and stricture are graded as absent (0) or present (1); exudates and furrows are graded as absent (0), mild (1), or severe (2); rings are graded as absent (0), mild (1), moderate (2), or severe (3) [79]. Typical endoscopic findings showing linear furrows, esophageal rings and mucosal fragility are presented in Figure 2 and Figure 3.

According to Müller et al., 9% of patients with Schatzki ring had also EoE, sometimes without typical features for EoE [80]. Esophageal strictures not observed in endoscopy may be visible on esophagography with barium contrast and/or in high-resolution impedance planimetry, which additionally may help to assess the degree of esophageal fibrosis [81]. Reduced distensibility of esophagus measured by the EndoFLIP system, a new endoluminal functional lumen imaging probe, corresponds with a higher risk of food impaction [72].

Patients with EoE require frequent endoscopy with esophageal tissue sampling to assess the effectiveness of elimination diets and pharmacological therapy, as well as long-term control of relapse leading to inflammation and fibrosis. Therefore, there is a need for new non-invasive methods allowing assessment of esophageal eosinophilia; some already exist, such as the esophageal string test or cytosponge, but require further evaluation [81,82].

There are also questionnaire-based tools used for assessment of symptoms in EoE like, e.g., the Adult Eosinophilic Esophagitis Activity Index PRO (EEsAI) that assesses behavioral adaptation and severity, frequency, duration of dysphagia triggered by eating foods of eight different consistencies [83,84], or The Dysphagia Symptom Questionnaire (DSQ), which includes questions about the presence of dysphagia, pain intensity, and methods alleviating dysphagia [85]. Dół formularza

Exclusion of other diseases with esophageal eosinophilia is necessary. Diseases with possible esophageal eosinophilia are, e.g., GERD, hypereosinophilic syndrome, infections, and drug hypersensitivity reactions.

## 4. Therapy of Eosinophilic Esophagitis

**Figure 4:**
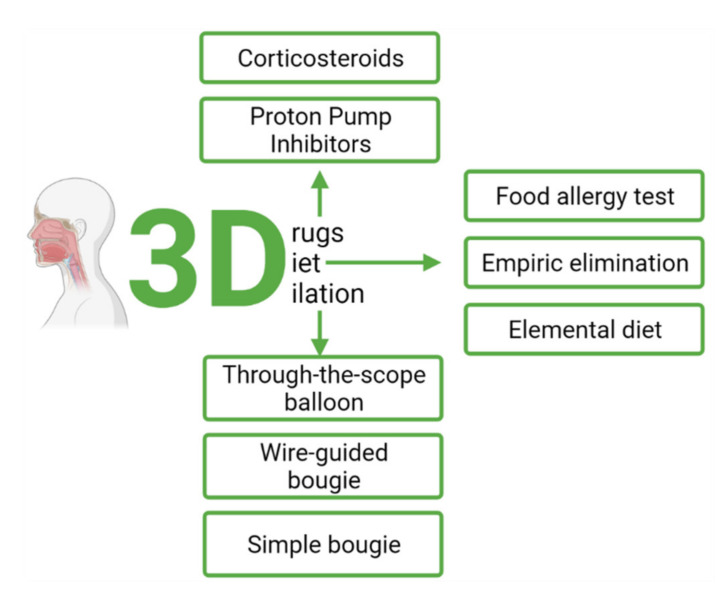
Treatment of eosinophilic esophagitis.

The main goal of EoE treatment is to eliminate inflammation by the removal of triggering factors and inhibition of inflammatory response by pharmacologic treatment [86]. Anti-inflammatory treatment aims to achieve deep remission seen in biopsy as lack of eosinophils and normal endoscopic esophagus appearance; often in clinical studies a peak eosinophil count below 5–6 per HPF is accepted as remission response. In the case of dysphagia, restoring proper food transition, along with proper nutritional status, is desired. Quality of life should also be addressed, as it is often impaired. Treatment of eosinophilic esophagitis is based on “3 D” therapy—drugs, diet, dilation (Figure 4). Since both drugs and mechanical procedures may cause side effects, it seems that early diagnosis with the implication of an effective diet is the safest therapeutic option. Unfortunately, diagnosis is often established late when stenosis occurs, and the only effective treatments are endoscopic procedures. On the other hand, in the case of severe symptoms due to inflammation or an ineffective elimination diet, the best option is the use of pharmacologic agents. The efficacy of any therapy should be assessed by endoscopy after a 6- to 12-week initial treatment.

### 4.1. Pharmacological Treatment

#### 4.1.1. Proton Pump Inhibitors (PPI)

Since esophageal eosinophilia responding to proton pump inhibitors (PPI-REE) is acknowledged as a subtype of EoE in some patients, high doses of PPI are effective. PPI are given twice daily for 8 weeks; recommended dose in adults is omeprazole 40–80 mg/d or equivalent; in children, 1–2 mg/kg/d of omeprazole or equivalent is recommended.

PPI may be effective in patients with EoE and GERD coexistence, relieving the reflux symptoms by gastric acid inhibition, but the additional effect is the improvement of epithelial barrier function and anti-inflammatory action, independent of their anti-secretory effect [87]. PPI are acid-activated weak bases, which by converting to sulfenic acid or sulfonamides inhibit gastric H,K-ATPases and block acid secretion [88]. However, PPI also block Th2 cytokine (Il-4, Il-13)-stimulated expression of eotaxin-3, through a non-gastric H+, K+ ATPase in esophageal squamous cells in patients with EoE. Eotaxin-3 is responsible for eosinophil chemoattraction to inflamed tissues [89,90]. In the study by Peterson et al., after 8 weeks of treatment with esomeprazole compared to swallowed fluticasone eosinophil, infiltration in esophageal tissue and symptoms of dysphagia decreased with treatment in both studied groups, without significant statistical difference between them [91,92]. According to the analysis of 23 observational studies, 42% of patients on PPI treatment due to esophageal eosinophilia achieved histological remission. Analyzed data were inconsistent and varied on criteria for patient selection, duration, and dose of PPI treatment, but it is still recommended to start with PPI treatment, which should be effective in a substantial group of patients including both adults and children [93]. Patients who responded to PPI treatment were less likely to develop diffuse esophageal narrowing [94]. Patients who do not respond to PPI treatment require the use of other pharmacological therapies [93].

#### 4.1.2. Corticosteroids

The most common treatment in patients unresponsive to PPI are topical corticosteroids. Topical steroids have 60% to 95% efficacy in histological remission after 2 months of treatment [9]. There are fluticasone propionate and budesonide formulations used in treatment. Since at the beginning of treatment there was no available preparation designed for esophageal deposition “asthma”, specific formulations have been used “off-label”. For children, budesonide solution with added sucralose to obtain viscous suspension is recommended in doses of 1–2 mg/d, and for adults 2–4 mg/d. Another possible way is to swallow aerosol during nebulization, but the effectiveness compared to viscous suspension is lower [95]. In adults, fluticasone from a metered-dose inhaler is used and the aerosol puffs should be swallowed not inhaled. For adults, the recommended fluticasone dose is 1760 mcg daily, and for children it is usually 880–1760 mcg/d [96]. It is advised to split doses, usually two puffs four times daily, with one before bedtime to prolong contact with esophageal mucosa. There is a high response to topical corticosteroids; according to eight double-blind placebo-controlled randomized control trials (RCTs), 65% of patients achieved histologic remission. The effectiveness depends on the formulation and the best efficacy in achieving histological remission has so far been budesonide in orodispersible tablets (BOT), approved by the European Medicines Agency in 2018. According to Lucendo et al.’s study, after 12 weeks of treatment with BOT given as induction therapy in dose 1mg twice daily, 85% of patients achieved remission [97]. There is also fluticasone propionate in orodispersible tablets under clinical study for efficacy and safety in subjects with EoE (no. NCT04281108). Side effects of topical steroids are mild local infections such as candidiasis, which resolves with typical anti-fungal treatment, and rarely systemic symptoms characteristic for use of higher doses of glucocorticoids such adrenal suppression or diminished growth. To prevent these complications, cortisol level should be monitored, especially in atopic children using additional corticosteroids for other allergic conditions.

Systemic corticosteroids should be restricted only for severe dysphagia or symptoms requiring fast withdrawal [9].

#### 4.1.3. Maintenance Therapy

It is known that discontinuation of treatment without elimination of triggering factors leads to relapse of EoE, typically in a 3–6 month period [98]. There are not enough studies to indicate consent recommendations for long-term therapy. Different strategies are under investigation and, so far, applied therapies have been individualized according to the patient’s response and expectations. Patients that responded to PPI therapy should continue the same type of treatment to maintain disease remission, but in half the dose used for induction. In a study by Gutiérrez-Junquera, 78% of children were in remission after one year of treatment on a 50% reduced dose of PPI [99]. Similar effects were observed in adults [100]. The use of topical corticosteroids in maintenance therapy is also effective with half the dose applied [96,101]. In a study by Strauman and al., efficacy of doses of 0.5 mg or 1 mg BOT given twice daily were compared in adults with EoE in maintaining remission for up to 48 weeks. At the end of treatment, in persistent remission were 73.5% of patients receiving BOT 0.5 mg and 75% receiving BOT 1.0 mg [102]. Since there are no recommendations on how to manage a patient with asymptomatic esophageal eosinophilia, clinical follow-up is reasonable due to possible fibrotic consequences [103].

#### 4.1.4. Emerging Therapies

The use of biological therapy is still under investigation. According to three RCTs, use of anti –IL-5 (mepolizumab and reslizumab) treatment did not induce remission in patients with EoE; similar results were found for infliximab.

Promising results with the decrease in esophageal eosinophilia and EoE symptoms were observed after the use of dupilumab—anti Il-4 and monoclonal antibody blocking Il-13 (RPC4046) [104]. Published case reports suggest the possible efficacy of vedolizumab [105].

Other drugs such as montelukast, antihistaminics, and cromolyn have not been proven to be effective [106,107,108,109]. Azathioprine, 6-mercaptopurine may be effective in maintenance therapy in corticosteroid-dependent patients with EoE, but there are only single case studies and scarce data concerning efficacy which does not allow recommendation of thiopurines, known for their potential serious side-effects. The effectiveness of other immunosuppressants, such as cyclosporine, tacrolimus, and methotrexate, has not been assessed in EoE [109].

### 4.2. Dietary Management

Dietary management is one of the essential non-pharmacological treatments of EoE in both adults and children. Several dietary approaches can be found in the current literature, and they focus mainly on elimination according to food allergy tests, empiric elimination, or elemental diet [110].

**Figure 5:**
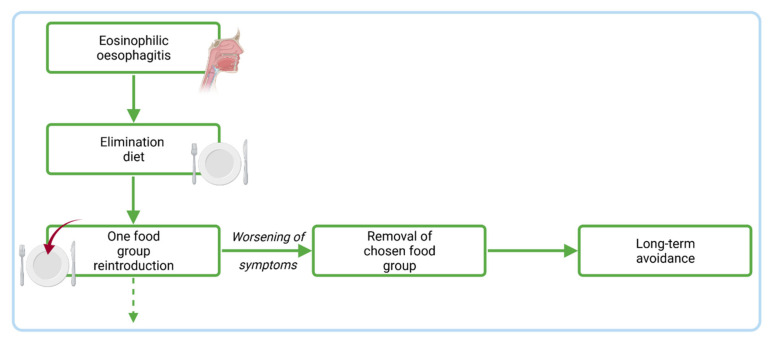
A simplified algorithm for an elimination diet in eosinophilic esophagitis.

**Figure 6:**
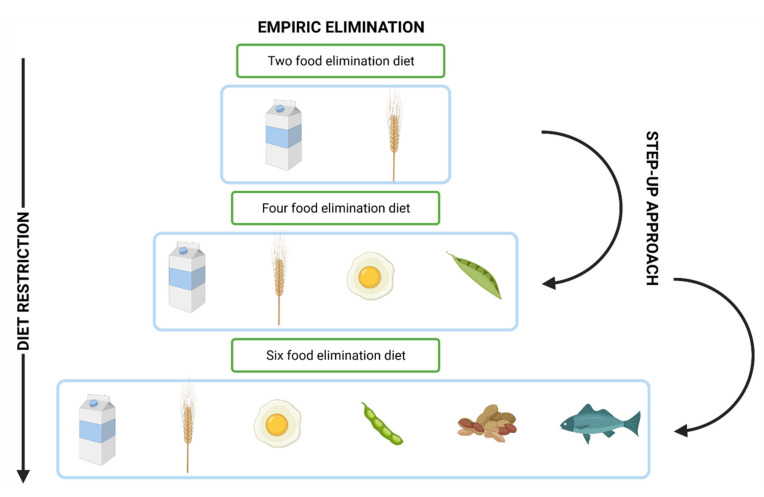
An empiric elimination diet—the six-food elimination diet (SFED).

To investigate if an elimination diet is effective and suitable for the patient, histological and clinical remission must be achieved [30]. Then, elimination should be conducted for around six weeks and slowly one group of food products should be introduced every six weeks. Any product that causes EoE should be removed from the diet permanently. A simplified algorithm for the elimination diet is presented in Figure 5. One of the empiric elimination diet approaches is based on the six groups of food products (the so-called six-food elimination diet, SFED) (Figure 6). This approach is usually very effective in EoE in all age groups; however, it should be noted that, the in studies of Gonsalves et al. and Lucendo et al., these six groups were constantly eliminated from the diet, which is advisable to maintain remission [111]. It is associated with a high level of dietary restriction and the number of clinical evaluations (e.g., endoscopies) can significantly increase, discouraging patients from following the diet. When the number of eliminated food groups is too high, another approach can be considered, which eliminates only four groups of food products (the so-called four-food elimination diet, FFED) (Figure 6); however, according to the available studies, its effectiveness can be lower than the previous approach (although it is was proven to be effective in 50–60% of the studied individuals) and then, more restrictive diets—for example SFED—can be considered. This approach, on the other hand, is called a step-up elimination diet, which eliminates only two food groups at the beginning (the so called two-food elimination diet, TFED) (Figure 6) and then eliminates the top four (FFED) and top six groups (SFED) [112]. Furthermore, elimination can be based on food allergy skin tests—the atopy patch test (ATP) or skin prick test (SPT). This approach can also be considered if the clinicians and patients want to possibly decrease the number of endoscopies. In the study of Arias et al., allergy testing-based food elimination diet led to remission in almost 50% of patients; however, the six-food elimination diet and elemental diet led to remission in 72% and 90.8% of cases, respectively [113]. Taking the current studies into the account, this approach is not recommended in EoE treatment. Another approach is an elemental diet consisting of free amino acids, which—although usually highly effective in EoE—is used in a limited way, mostly due to the high costs, low adherence, low palatability, and the need (usually) for a nasogastric tube in younger patients [114]. It should be also remembered that the reintroduction of food products should be longer. This approach can be considered if the patient is not willing to follow a more restricted diet or had a severe EoE course [115].

In summary, elimination diets are an effective treatment for EoE; however, each approach has its limitations. Elemental formulas are highly effective but may not be the most appropriate long-term option. Skin allergy tests are less effective and are not generally recommended but empiric eliminations are more effective and easier to follow. Nevertheless, long-term efficacy and adherence should be investigated further regardless of the chosen approach, also in comparison with pharmacological treatment [116,117].

### 4.3. Endoscopic Treatment

The therapeutic approach in eosinophilic esophagitis (EoE) consists of the “3D” concept: diet, drugs, and dilation [118]. Due to difficulty in evaluating the symptoms objectively and uniformly, as patients tend to modify their dietary and eating behavior to avoid dysphagia or impaction of food, a histological improvement from endoscopic biopsies is usually used as the primary outcome of treatment rather than symptomatic improvement. While it is not a formal recommendation or a guideline, the use of repeated esophagogastroduodenoscopy (EGD) with biopsy to assess disease activity after initial treatment or after a change in therapy is reasonable [119]. After 6–12 weeks of the initial treatment or when the change in therapy is made, the effectiveness of the treatment should be assessed by endoscopy. At the moment, a threshold of <15 eos/hpf from upper endoscopy biopsies to define an adequate therapeutic response serves as a response criterion until a better measure is established [120]. According to the Clinical Practice Guidelines of AGA, the recommended frequency for EGD with biopsy during clinical follow-up is identified as a knowledge gap and currently depends on the clinician’s decision and on the severity of the initial clinical presentation of the patient [119].

Dilation therapy is recommended for symptomatic patients with EoE who end up having esophageal stricture or narrowing despite medical therapy. Three types of procedures have primarily been used, (1) through-the-scope (TTS) balloon dilation; (2) the wire-guided bougie dilation; and (3) the simple bougie dilation (Maloney and Hurst-Style)—they are the only endoscopic treatment methods available for EoE. Endoscopic dilation should be performed in all patients with fibrostenotic abnormalities (including an esophagus with a diameter < 13 mm) that cause dysphagia or food impaction, despite the use of dietary or pharmacological treatment. Endoscopic dilation should not be the only intervention used since it has no effect and does not control chronic esophageal inflammation that contributes to esophageal remodeling and formation of fibrotic strictures. In cases of severe and symptomatic esophageal stricture, dilation together with concomitant anti-inflammatory treatment (PPI, topical corticosteroids or diet) may quickly achieve clinical, endoscopic and histological remission of EoE [120,121].

In the study by Moawad F.J. et al., the effectiveness of dilation has been reviewed in a meta-analysis of 27 observational studies involving a total of 845 patients and 1820 dilations were considered. Symptomatic improvement was documented in 95% of patients, with a highly variable duration of symptomatic relief. Complications were uncommon and they included perforation (0.38% of cases), hemorrhage (0.05%) and hospitalization (0.67%) and no deaths have been reported [121,122]. Patients requiring dilation are primarily adult patients since esophageal stricture and narrowing develop progressively during long-term and persistent eosinophilic inflammation [123]. Younger age patients with EoE needing multiple dilations, patients with upper esophageal strictures, and those in whom stricture cannot be passed with an endoscope are at risk for dilation-related unwanted events [124]. In a study conducted by Runge et al., the TTS methods exhibited the potential to extend the esophagus lumen further than the bougie method. No significant differences were reported regarding complications. In patients with EoE, it is important for the endoscopist to gradually and gently dilate because chest pain or mucosal tears can often occur secondarily to esophageal mucosal fragility [125,126].

## 5. Summary—New Perspective and Conclusions

EoE is one of the gastrointestinal entities, along with inflammatory and allergic diseases, that have been occurring more frequently among children, adolescents and adults for recent years, probably as an effect of environmental changes in patients with genetic susceptibility. Since the course of the EoE may be asymptomatic or symptoms may not be specific at the beginning, to prevent further complications it is important to find the groups with increased risk and actively search for the signs of EoE, such as, e.g., in children with food allergies or pollen allergy individuals with abdominal pain occurring during the pollen season. Thus far, only endoscopic esophageal mucosa sampling with histopathologic assessment may confirm diagnosis and control therapy efficacy. Recognition of triggering food allergens is based usually on empiric food elimination diet with subsequent withdrawal or introduction of new food according to histopathologic assessment. This approach is time-consuming and requires invasive procedures influencing patients’ quality of life. Low quality of life is also a consequence of symptoms such as food impaction leading to altered eating behaviors and avoidance of social food consumption. These problems generate others such as malnutrition developed by choosing a smaller number of types of easily digested food items often in small quantities.

There are many challenges in the management of EoE. First of all, it is essential to find diagnostic tools showing the exact food allergens that need to be eliminated in the diet, because this would remove the need for pharmacologic treatment in many patients. It is important to establish the frequency of esophageal mucosa monitoring to prevent fibrosis and strictures to implement new non-invasive methods of disease activity monitoring, such as, e.g., saliva, stool sampling, or esophageal sampling without endoscopy, to distinguish the patients more prone to fibrosis that need more aggressive therapy, e.g., by genetic testing. Treatment is another problematic issue, since steroids are the most potent drugs in resolving eosinophilic inflammation available for most patients, but side effects and unknown safe treatment duration are limiting their extended use. There are new drugs, mainly biological therapies, that are possibly effective in EoE treatment, but clinical studies on a sufficient group of patients must confirm their safety and efficacy. The promising alternative would be targeted alteration of human microbiota preventing or reversing the development of allergic diseases, and consequently EoE.

We believe that the analysis of molecular mechanisms involved in the pathogenesis of EoE could significantly facilitate the approach to the classification of the disease and its differentiation from, for example, GERD, as well as individualization of the therapy and treatment methodology. As of today, no therapy based on molecular mechanisms improving the condition of EoE has been established, which suggests the need to formulate translational research to select and optimize treatment methods. Perhaps current EoE clinical trials should include transcriptional analysis of selected EoE-determinant genes such as *CCL26* and *FLG* to develop optimal therapy and treatment selection.

## Figures and Tables

**Table 1:** Eosinophilic esophagitis expression profile. Description of genes conditioning the eosinophilic esophagitis genome-wide association studies. An increase in expression level is indicated by a green arrow, a decrease by a red arrow. Chromosome location as described by the GeneCards database (GRCh38/hg38).

Gene	Location	Expression in EoE	Impact on EoE	Cell Type
*TSLP* *Thymic Stromal Lymphopoietin*	chr5:111,070,062–111,078,026	↑	increased Th2 responses induce Th2 cell development and activate eosinophils and basophils	esophageal epithelial cells
*CCL26* *C-C Motif Chemokine Ligand 26*	chr7:75,769,524–75,791,597	↑	eosinophil recruitment	esophageal epithelial cells
*TGFB1* *Transforming Growth Factor Beta 1*	chr19:41,301,587–41,353,922	↑	response to steroid, eosinophil adhesion, esophageal remodeling	eosinophils and mast cells
*CRLF2* *Cytokine Receptor Like Factor 2 2*	chrX:1,190,437–1,212,762	Lack of data	increased Th2 responses	eosinophils, mast cells, dendric cells, basophils
*WDR36* *WD Repeat Domain 36*	chr5:111,091,716–111,130,502	unchanged	induces Th2 cell development and activates eosinophils and basophils	esophageal epithelial cells
*STAT6* *Signal Transducer And Activator Of Transcription 6*	chr12:57,095,408–57,132,139	↑	primary mediator of IL-4 and IL-13 signaling, the downstream signaling mediator of IL-4Ralpha	eosinophils
*GATA-3* *GATA Binding Protein 3*	chr10:8,045,378–8,075,198	↑	transcriptional regulators that drive differentiation of Th0 CD4^+^ lymphocytes to Th2 lineages	esophageal epithelial cells
*TBX21* *T-Box Transcription Factor 21*	chr17:47,733,236–47,746,122	↑	transcriptional regulators that drive differentiation of Th0 CD4^+^ lymphocytes to Th1 lineages	esophageal epithelial cells
*FLG* Filaggrin	chr1:152,302,165–152,325,239	↓	Reduced barrier, increased sensitization	unknown
*Il-13* *Interleukin 13*	chr5:132,656,263–132,661,110	↑	responsible for pathophysiological changes in the esophageal epithelium	esophageal epithelial cells
*Il-5* *Interleukin 5*	chr5:132,539,194–132,556,864	↑	major role in eosinophil-related disorders, eosinophil differentiation factor, and activator	esophageal epithelial cells
*Il-4* *Interleukin 4*	chr5:132,673,986–132,682,678	↑	initiating the Th2 response through differentiation of naive T helper cells into Th2 type cells	esophageal epithelial cells
*MUC5AC* *Mucin 5AC, Oligomeric Mucus/Gel-Forming*	chr11:1,157,953–1,201,138	↑	protects the mucosa from infection and chemical damage	esophageal epithelial cells
*SMAD2* *SMAD Family Member 2*	chr18:47,808,957–47,931,188 and	↑	associated with the recruitment of eosinophils	eosinophils, esophageal epithelial cells mast cells
*SMAD3* *SMAD Family Member 3*	chr15:67,063,763–67,195,195	↑	associated with the recruitment of eosinophils	eosinophils, esophageal epithelial cells mast cells
*VCAM1* *vascular cell adhesion molecule*	chr1:100,719,742–100,739,045	↑	increased inflammatory cell binding to the endothelium of esophageal vessels, facilitating infiltration of eosinophils into the esophagus	eosinophils, mast cells, endothelial cells
*CAPN14* *Calpain 14*	chr2:31,173,056–31,233,970	↑	Induced by Il-13, involved in epithelial homeostasis and repair, possesses STAT6 binding sites	esophageal epithelial cells
*ICAM1* *intracellular adhesion molecule 1*	chr19:10,271,093–10,286,615	↑	implicated in the tissue recruitment of eosinophils and mast cells	eosinophils, mast cells, endothelial cells

## Data Availability

Data are available in publicly accessible databases. The data presented in this study are openly available in the Medline and PubMed databases and on the publisher’s website. The keywords that were used: “eosinophilic esophagitis”, ”epidemiology”, food allergy”,” microbiota”, “immunogenetics”, “treatment”, “environmental factors”, ”novel therapy”, ”guidelines” “histopathology”, ”genetic factors”; “diet”; “elimination diet”; “dietary management”. All data in the text are quoted and all works used are listed in the bibliography along with the DOI and reference numbers.
